# Retraction: Long non-coding RNA ANRIL alleviates H2O2-induced injury by up-regulating microRNA-21 in human lens epithelial cellss

**DOI:** 10.18632/aging.205115

**Published:** 2023-10-14

**Authors:** Shanshan Du, Jingzhi Shao, Ying Qi, Xuhui Liu, Jingjing Liu, Fengyan Zhang

**Affiliations:** 1 Department of Ophthalmology, The First Affiliated Hospital of Zhengzhou University, Zhengzho, Henan 450052, China

**Keywords:** cataract, H_2_O_2_, human lens epithelial cells, lncRNA ANRIL, microRNA-21

**This article has been retracted:** Aging has completed its investigation of this paper after communicating with the authors. We received a letter from the authors stating that, “it was found in our subsequent studies that the AMPK pathway in lens epithelial cells was not activated after H_2_0_2_ intervention. In order to better investigate the mechanism of oxidative stress in lens epithelial cells and explore new signaling pathways, we plan to withdraw/retract the manuscript and redesign the research.” All authors agreed with this decision and signed the letter.

